# Bilateral Internal Carotid Artery Dissection as a Possible Complication in a patient with Covid-19 infections and coughing

**DOI:** 10.22088/cjim.13.0.281

**Published:** 2022

**Authors:** Mohammad Ghorbani, Abolghasem Mortazavi, Ghazwan Lafta, Mahdi Kadkhodazadeh Asl, Reza Bahrami, Farhad Rahbarian

**Affiliations:** 1 Division of vascular and Endovascular Neurosurgery, Firoozgar Hospital, Iran University of Medical Sciences, Tehran, Iran; 2 Department of Surgery, University of Al-Ameed, Karbala, Iraq

**Keywords:** Carotid stenting, Cough, dissection, Stroke, COVID-19

## Abstract

**Background:**

Cervical carotid dissection is one of the causes of ischemic stroke in young people. Most of the patients with carotid dissection do not have connective tissue diseases (Marfan syndrome, Ehlers-Danlos syndrome). It seems that dissection may occur without an obvious cause or may follow environmental injuries like vigorous neck movements, chiropractic manipulation, emesis, severe coughs, and some infections. We present a case of bilateral carotid dissection in a patient following coronavirus infection and severe coughs.

**Case Presentation:**

A 38-year-old right-handed man presented with recurrent episodes of transient right hemiparesis and aphasia. He had a history of coronavirus infection and severe persistent, nonproductive cough 7 days before the onset of his symptoms. Carotid angiography showed tapered flame-like appearance in proximal segment of left ICA starting about 2 cm distal to the carotid bulb caused complete occlusion of left ICA and in right CCA angiography there is pseudo aneurysm in right cervical ICA just before the Petrous segment. In 3 months in follow up DSA there is evidence of complete occlusion of right pseudo aneurysm and recanalization of left ICA without stenosis.

**Conclusion:**

COVID-19 may have role in the processes that eventually led to CAD

Cervical carotid dissection (CAD) is one of the causes of ischemic stroke in young people (1). According to the literature, the odds of a stroke secondary to dissection are up to 20% (2). However increasingly diagnosed by modern radiological techniques, its pathogenesis remains unclear. Most of the subjects with carotid dissection do not have connective tissue diseases (Marfan syndrome, Ehlers-Danlos syndrome). It seems that dissection may occur without an obvious cause or may follow environmental injuries like vigorous neck movements; chiropractic manipulation, sneezing, emesis, and some infections (3-5). We present a case of bilateral carotid dissection in a patient following coronavirus infection and severe coughs.

## Case presentation

A 38-year-old right-handed man presented to our center complaining of recurrent episodes of transient right hemiparesis and aphasia. The patient had a history of coronavirus infection and severe persistent, nonproductive cough 7 days before the onset of his symptoms. The right-sided symptoms had been occurring two times, each time lasting 10 minutes. 

**Figure1:**
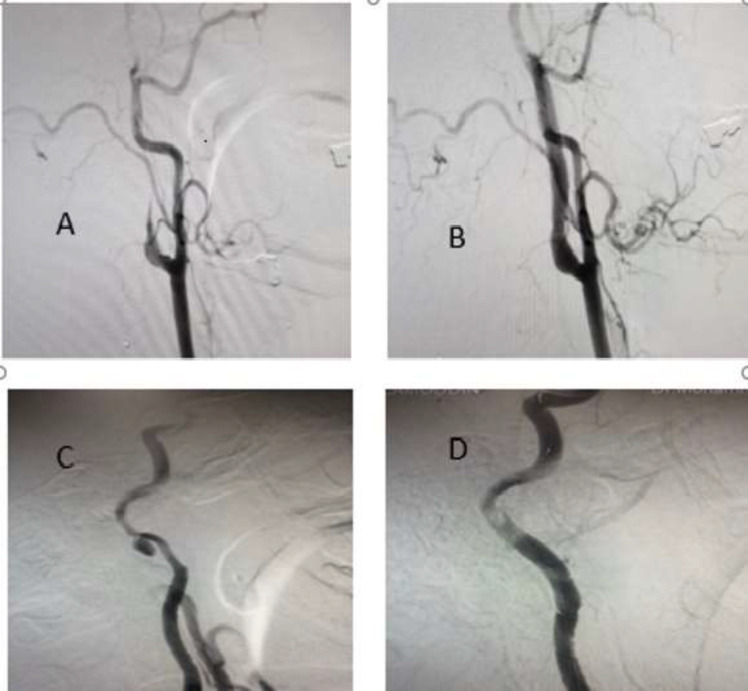
Cervical carotid angiography: (A) Left carotid angiography (lateral view) reveals the dissected part of proximal ICA. (B) Left-side post-stenting angiography (Lateral view). (C) Right carotid angiography (oblique view) shows the pseudo-aneurysm in cervical portion. (D) Right-side post-intervention angiography (lateral view)

His medical history was negative for hypertension, and smoking or recent trauma. On physical examination, the patient was normotensive, intact neurological examination, and no carotid bruits were detectable. Bilateral peripheral pulses were normal and equal. Routine laboratory tests were normal. A computed tomographic (CT) scan and magnetic resonance imaging of the brain was normal. The patient with diagnosis of transient ischemic attack (TIA) was immediately treated with intravenous heparin and oral antiplatelet agents. Carotid angiography showed tapered flame-like appearance in cervical portion of left internal carotid artery (ICA) starting about 2 cm distal to the carotid bulb caused complete occlusion of left ICA (Figure 1-A). These findings confirmed the diagnosis of ICA dissection. In right common carotid artery (CCA) angiography there was pseudo aneurysm in right cervical ICA just before the petrous segment. (Figure1-C)

Left vertebral artery angiography revealed collateral circulation to bilateral ICA from bilateral posterior communicating arteries. We decided to treat the patient with simultaneous bilateral carotid artery stenting as he was symptomatic despite receiving medical treatment. A femoral artery approach was obtained and selective bilateral carotid angiogram was performed. The 8F guiding catheter was put to the left CCA and the PILOT 150 micro-guidewire was passed from occluded segment of left ICA after that (8×40 mm) self-expanding carotid stent (Boston Scientific, Marlborough, MA, USA) was deployed in the ICA. Post-stenting angioplasty was performed with inflation (5×20 mm) NC balloon (Medtronic). We decided to put a stent in right ICA. There was a pseudo-aneurysm in distal cervical portion of ICA. The 8F guiding catheter was introduced to the right CCA and the lesion was crossed by PILOT 150 micro-guidewire and the stent was deployed in the distal cervical portion of ICA.There were no neurological complications. After 3 months in follow up catheter angiography there is evidence of complete occlusion of right pseudo aneurysm and recanalization of left ICA without stenosis (Figure 1-B and 1-D).

## Discussion

The patient introduced in this case report had spontaneous bilateral cervical carotid dissection with frequent TIA symptoms. It is generally believed that spontaneous dissection occurs in the context of underlying vascular wall structure disorders although no definite arteriopathy is found in many cases (1). Congenital connective tissue diseases may be detected in 1-5% of the patients and 5% have a positive history of a connective tissue disease in a family member (6). Our patient neither had an affected family member nor was he affected. 

A positive history of a recent coronavirus infection may be a risk factor for vascular dissection (3). Two prospective studies found a higher prevalence of infection in patients with stroke secondary to dissection compared to patients with stroke due to other causes (3, 4). There is a case report of carotid dissection following severe coughs in an 8-year-old child with no risk factors (2). 

Some studies found an association between pertussis and CAD. Although difficult to prove, it seems that severe coughs due to pertussis play a role in dissection (7, 8). According to several studies, causing diffuse endothelial inflammation radicals and inflammatory factors increase following infection, resulting in injury to the extracellular matrix and causing diffuse endothelial inflammation. In addition, infections may be associated with a number of mechanical factors, including severe vomiting, coughing, and sneezing, which could trigger dissection (3). There are reports of eosinophilic infiltration in autopsy specimens of spontaneous coronary artery dissection (9); however, because carotid dissection usually improves spontaneously and has a mortality of less than 5%, there may not be enough autopsy studies about this entity (1).

 Bilateral cervical ICA dissection with sudden onset more probably compromise hemodynamic than atherosclerotic lesions because the collateral circulation has not sufficiently developed. The indication of carotid artery stenting (CAS) for carotid artery dissection is the following situations: (a) symptoms persist or worsen under medical therapy; (b) significant hemodynamic compromise or (c) There is contraindications for antithrombotic therapy (10). Our patient had the first criterion mentioned above. In conclusion COVID-19 may have role in the processes that eventually led to CAD. Dissection may occur following a minor trauma as severe coughs, or recent infection. Clinical suspicion of CAD in a young patient with stroke is very important to prevent further cerebral damage through triage and treatment. 
